# Genotoxicity of 12 Mycotoxins by the SOS/umu Test: Comparison of Liver and Kidney S9 Fraction

**DOI:** 10.3390/toxins14060400

**Published:** 2022-06-10

**Authors:** Maria Alonso-Jauregui, Elena González-Peñas, Adela López de Cerain, Ariane Vettorazzi

**Affiliations:** 1 MITOX Research Group, Department of Pharmacology and Toxicology, School of Pharmacy and Nutrition, Universidad de Navarra, 31008 Pamplona, Spain; malonso.17@alumni.unav.es (M.A.-J.); acerain@unav.es (A.L.d.C.); 2 MITOX Research Group, Department of Pharmaceutical Technology and Chemistry, School of Pharmacy and Nutrition, Universidad de Navarra, 31008 Pamplona, Spain; mgpenas@unav.es

**Keywords:** genotoxicity, liver S9, kidney S9, in vitro, bioactivation

## Abstract

Liver S9 fraction is usually employed in mutagenicity/genotoxicity in vitro assays, but some genotoxic compounds may need another type of bioactivation. In the present work, an alternative S9 fraction from the kidneys was used for the genotoxicity assessment of 12 mycotoxins with the SOS/umu test. The results were compared with liver S9 fraction, and 2–4 independent experiments were performed with each mycotoxin. The expected results were obtained with positive controls (4-nitroquinoline-N-oxide and 2-aminoanthracene) without metabolic activation or with liver S9, but a potent dose-dependent effect with 4-nitroquinoline-N-oxide and no activity of 2-aminoanthracene with kidney S9 were noticed. Aflatoxin B1 was genotoxic with metabolic activation, the effect being greater with liver S9. Sterigmatocystin was clearly genotoxic with liver S9 but equivocal with kidney S9. Ochratoxin A, zearalenone and fumonisin B1 were negative in all conditions. Trichothecenes were negative, except for nivalenol, 3-acetyldeoxynivalenol, 15-acetyldeoxynivalenol, T-2 and HT-2 toxins, which showed equivocal results with kidney S9 because a clear dose-response effect was not observed. Most of the mycotoxins have been assessed with kidney S9 and the SOS/umu test for the first time here. The results with the positive controls and the mycotoxins confirm that the organ used for the S9 fraction preparation has an influence on the genotoxic activity of some compounds.

## 1. Introduction

Mycotoxins are secondary metabolites produced by fungi with several toxicological effects to humans and animals [1]. Genotoxicity and carcinogenicity are important toxicological endpoints due to the possible chronic exposure to mycotoxins [2]. A prioritization strategy based on the genotoxic potential of 12 mycotoxins has been performed using in silico approaches and the SOS/umu test [3]. The mycotoxins selected were aflatoxin B1 (AFB1), sterigmatocystin (STER), ochratoxin A (OTA)—all of which are produced by *Aspergillus* and *Penicillium* species—and 9 mycotoxins produced by *Fusarium* species: zearalenone (ZEA), fumonisin B1 (FB1), nivalenol (NIV), deoxynivalenol (DON), 3-acetyldeoxynivalenol (3ADON), 15-acetyldeoxynivalenol (15ADON), T-2 (T-2), HT-2 (HT-2) and fusarenon-X (F-X). In the SOS/umu test performed with and without metabolic activation, AFB1 and STER were classified as genotoxic dependent on metabolic activation, whereas OTA, ZEA and FB1 were nongenotoxic [3]. The S9 mixture for the SOS/umu test was prepared with a commercial post-mitochondrial supernatant 9000 g fraction (S9) from rodent livers at 8%.

The study design of the in vitro genotoxicity assays must consider the use of an exogenous metabolic activation fraction for chemical testing [4,5]. Rat liver S9 fraction is the standardly used in the genotoxicity assays; it can be obtained from different rat strains and after different treatments with enzyme inducers, but some inconsistencies have been found [6]. Moreover, there is no a clear statement on the rat strain, enzyme concentration (mg protein/mL) and the most convenient treatment for the induction. This lack of homogeneity can generate variable genotoxic responses during in vitro assessments [6]. To overcome this problem, some alternatives have been proposed, such as the biotechnological animal free “ewoS9R” [7]. On the other hand, the International Council for Harmonisation of Technical Requirements for Pharmaceuticals for Human Use (ICH) guideline for the genotoxicity assessment of pharmaceuticals (ICH S2R1) recommends the use of alternative methods/systems for metabolic activation when negative results are obtained in vitro and further testing is considered [5]. One of the reasons for explaining the failure of these strategies for detecting genotoxic carcinogens is concerning metabolism and the different biotransformation pathways of chemicals in cells of different tissues. Therefore, the International Workshop on Genotoxicity Test (IWGT) Strategy Expert Group also recommended the use of alternative systems/tissues/species for genotoxicity testing in vitro [8]. The Organization for Economy and Co-operation and Development (OECD) bacterial reverse mutation assay guideline also considers different patterns for the metabolic activation of chemicals as a cause for missing some mutagens with this assay [4]. Different organs than liver have been considered for the S9 preparation (e.g., kidney and spleen) [9]. Human kidney microsomes exerted a similar response to a liver S9 fraction in the biotransformation of peptides [10]. The kidney has a prominent role in the toxicity of numerous drugs, environmental pollutants and natural substances [11]. In addition, several organic substances are nephrotoxic just after being transported into tubular cells. For instance, citrinin induces proximal tubular necrosis [12]. OTA has been shown to be negative in mutagenicity assays [13]. However, it was mutagenic when using a mice kidney microsomal fraction [14]. Therefore, the use of a kidney S9 fraction in genetic toxicology testing may be relevant for some compounds.

In this work, the in vitro genotoxic effect of 12 mycotoxins and the influence of the tissue origin of the metabolic activation fraction has been studied using two S9 fractions. On the one hand, is the standard liver S9 fraction from induced rats and on the other, is a kidney S9 fraction from uninduced rats. For that purpose, the medium-throughput test SOS/umu, previously used to prioritize mycotoxins based on their genotoxicity [3], has been selected. It has been demonstrated to have a high concordance with the regulatory accepted Ames test (TG OECD 471 [4]) [15,16]. The SOS/umu was selected because it has the following advantages with respect to the Ames test: (i) the results are obtained the same day of the experiment (1 day); (ii) the amount of test compound is lower; and (iii) it allows for the assessment of a maximum of six different compounds in one experiment. In summary, there is a significant reduction in material expenses and workload [17]. In the present study, the SOS/umu test has been performed with each of the mycotoxins and with both S9 fractions in 2–4 independent assays. Negative and positive controls were included in all the assays.

## 2. Results

**Figure 1:**
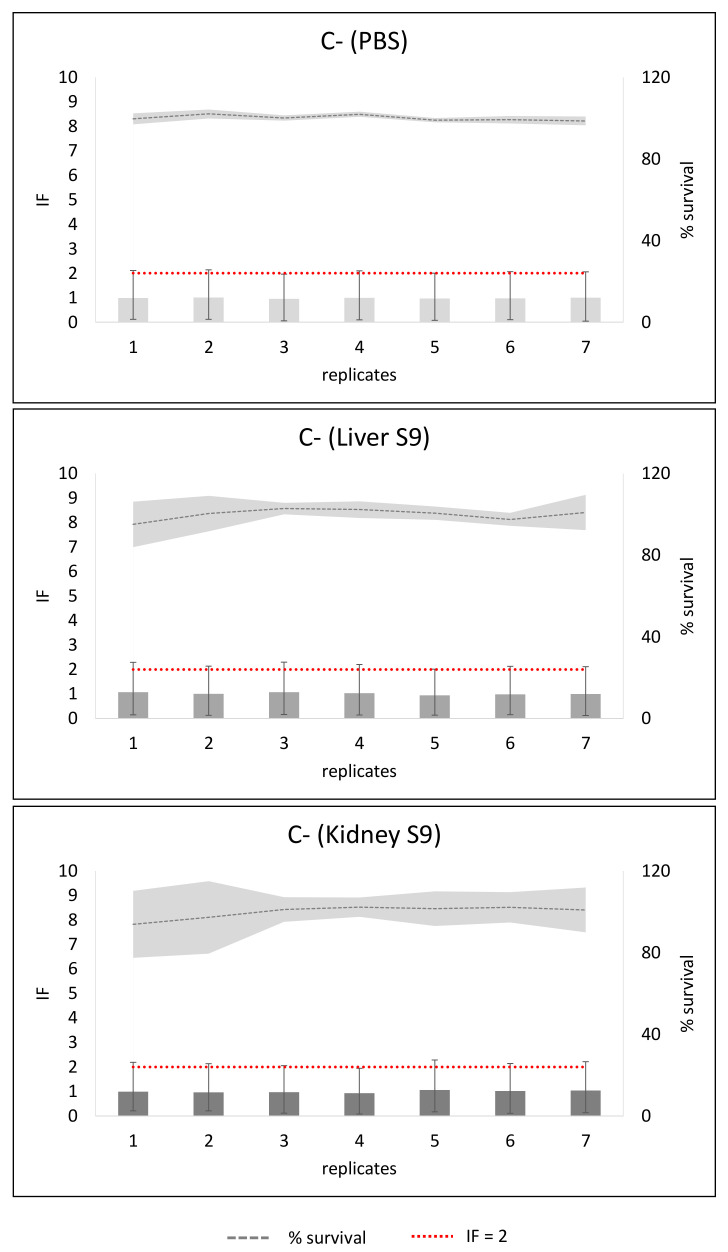
Negative control results in the SOS/umu test without metabolic activation (PBS) or with metabolic activation from liver S9 or kidney S9. Average and standard deviation of each of 7 replicates per experiment from a total of 8 experiments are presented. The bars represent the induction factor (IF) and the grey line the bacterial survival as percentage. The standard deviation (SD) between experiments is presented with the SD bars or with the soft grey area for the survival. Results were considered non-toxic if survival was >80%. Values were considered genotoxic if inductor factor (IF) value was ≥2 at non-toxic concentrations. The red line has been depicted to indicate IF = 2.

The results of the negative control (C−) from eight independent experiments are presented in Figure 1. Seven replicates were generated in each experiment. The average and standard deviation of each of the replicates are shown in the figure. The absence of toxicity and genotoxicity is confirmed in each condition.

**Figure 2:**
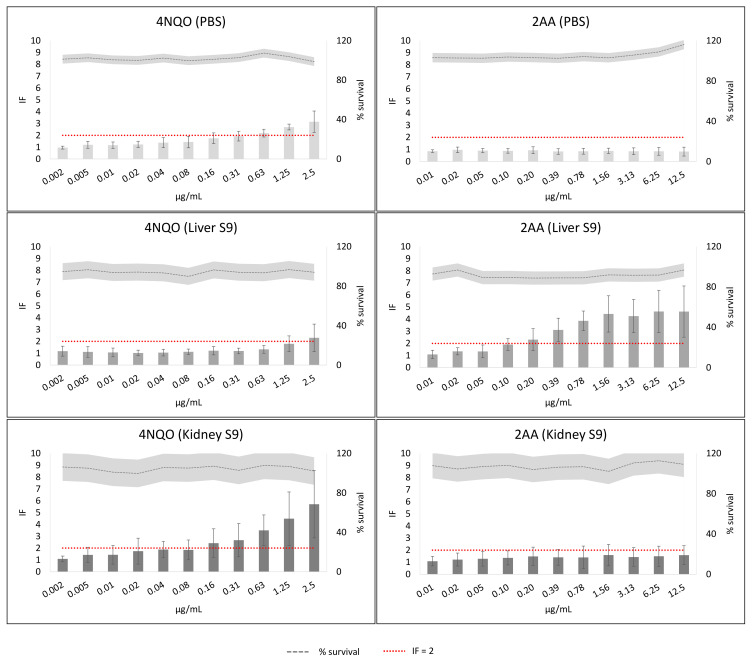
Positive controls results of the SOS/umu test without metabolic activation (PBS) or with metabolic activation from liver S9 or kidney S9. Average and standard deviation from 8 independent experiments are presented. The bars represent the inductor factor (IF) and the grey line the bacterial survival as percentage. The standard deviation (SD) is presented with the SD bars or with the soft grey area for the survival. Concentrations were considered non-toxic if survival was >80%. A compound was considered genotoxic if inductor factor (IF) value was ≥2 at non-toxic concentrations. The red line has been depicted to indicate IF = 2.

The results of the positive controls 4-nitroquinoline-N-oxide (4NQO) and 2-aminoanthracene (2AA) without metabolic activation (PBS) and with metabolic activation from liver (liver S9) or from kidney (kidney S9) are presented in Figure 2. 4NQO without metabolic activation has IF values greater than 2 at high concentrations (0.63–2.5 µg/mL) with a concentration–response tendency. In the presence of liver S9, a positive result was only observed at the highest concentration (2.5 µg/mL), but with kidney S9, a strong genotoxic effect with a concentration–response trend was registered. Moreover, the highest concentration had IF values around 6 (Figure 2). IF variability was greater with kidney S9 (max % CV: 62.9) than with liver S9 (max % CV: 50.6). Considering 2AA, it was negative without metabolic activation and with kidney S9. However, it was positive with liver S9, showing a concentration–response tendency and reaching IF values around 4 (Figure 2). IF variability was greater with kidney S9 (max % CV: 66.7) than with liver S9 (max % CV: 45.8).

**Figure 3:**
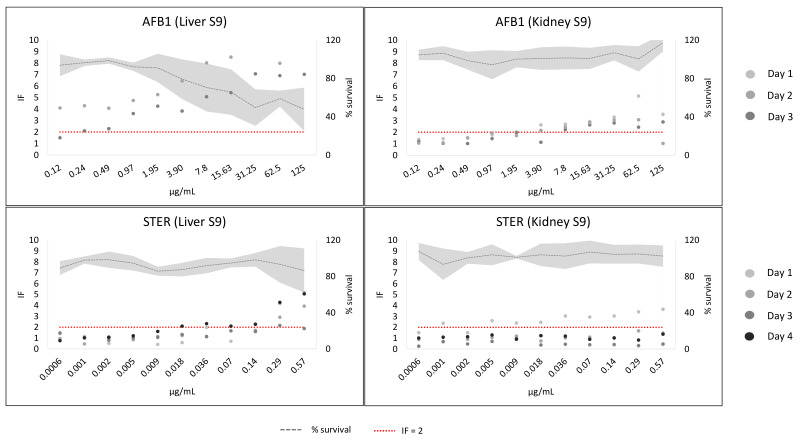
AFB1 and STER results in the SOS/umu test with metabolic activation from liver S9 or kidney S9. The number of independent experiments of AFB1 are 2 for the liver S9 or 3 for the kidney S9. The number of independent experiments of STER are 4 in both conditions. The dots represent the inductor factor (IF) of each individual experiment and the grey line the mean bacterial survival as percentage. The standard deviation of the survival is presented with the soft grey area. Concentrations were considered non-toxic if survival was >80%. A compound was considered genotoxic if inductor factor (IF) value was ≥2 at non-toxic concentrations. The red line has been depicted to indicate IF = 2. Data from two assays with liver S9 (days 3 (AFB1) and 4 (STER)) were published in [3]. Percentage survival values above 120 were corrected to 120%.

The results of AFB1 and STER are shown in Figure 3 and Figure S1 of Supplementary Materials. There was a weak genotoxic tendency without metabolic activation for both mycotoxins (see Figure S1). AFB1 showed a clear concentration–response tendency with both metabolic fractions, although the effect was more pronounced with liver S9. The bacterial survival decreases at the highest concentrations. However, AFB1 is still genotoxic and non-toxic at the lowest concentrations (see Figure 3). IF variability was greater with liver S9 (max % CV: 65.6) than with kidney S9 (max % CV: 39.9). STER gave IF values greater than two, and a concentration–response was evident with liver S9. However, equivocal results were observed with kidney S9: no genotoxicity in three out of four experiments. IF values of day 1 were above two with a weak concentration–response (see Figure 3). IF variability was greater with kidney S9 (max % CV: 87.2) than with liver S9 (max % CV: 47.4).

The results of DON, F-X, NIV, 3ADON, 15ADON, T-2 and HT-2 without metabolic activation were all negative (see Figures S2 and S4 of Supplementary Materials). The results with metabolic activation are shown in Figure S3 (DON, F-X) and 4 (NIV, 3ADON, 15ADON, T-2, HT-2). DON and F-X were negative with metabolic activation. The highest IF values obtained were 1.94 for DON (kidney S9, 431 µg/mL) and 1.88 for F-X (kidney S9, 1 µg/mL). There was no concentration–response tendency for any mycotoxin.

**Figure 4:**
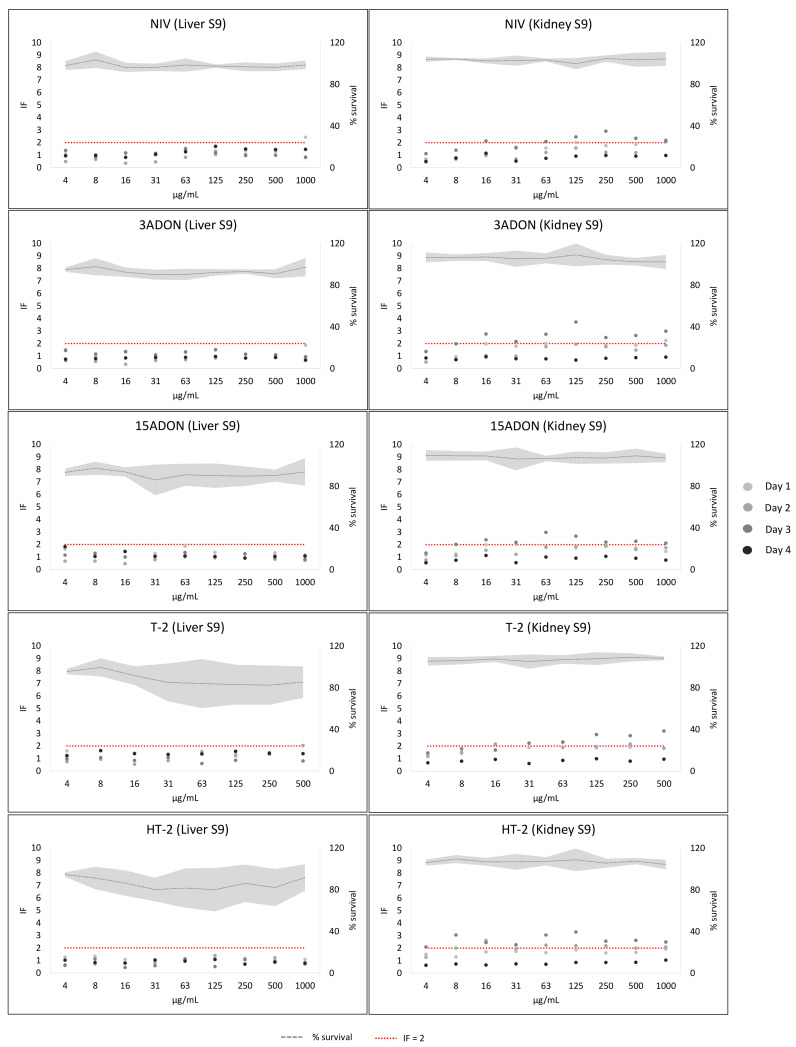
NIV, 3ADON, 15ADON, T-2 and HT-2 results in the SOS/umu test with metabolic activation from liver S9 or kidney S9. The number of independent experiments was 4 for all conditions. The dots represent the inductor factor (IF) of each individual experiment and the grey line the mean bacterial survival as percentage. The standard deviation of the survival is presented with the soft grey area. Concentrations were considered non-toxic if survival was >80%. A compound was considered genotoxic if inductor factor (IF) value was ≥2 at non-toxic concentrations. The red line has been depicted to indicate IF = 2. Data from two assays with liver S9 (days 2 and 3) were published in [3].

NIV, 3ADON, 15ADON, T-2 and HT-2 were all negative with liver S9 (Figure 4). However, NIV, 3ADON and T-2 gave IF values above two in three out of four experiments, and 15 ADON and HT-2 gave IF values above two in two out of four experiments with kidney S9. Nonetheless, there was not a clear concentration–response in any of the experiments (see Figure 4). IF variability was greater with kidney S9 (max % CV of 3ADON: 60.1; 15ADON: 51.8; T-2: 47.3; HT-2: 54.9) than with liver S9 (max % CV of 3ADON: 49.3; 15ADON: 42.8; T-2: 40.7; HT-2: 36). For NIV, liver S9 variability was greater (max % CV 54.2) than with kidney S9 (max % CV 51.3).

The results of OTA, ZEA and FB1 are shown in Figures S5 and S6 of Supplementary Materials. All the mycotoxins were negative both with liver or kidney S9 and without metabolic activation.

## 3. Discussion

Metabolic activation is a pivotal aspect in genetic toxicology testing, due to metabolic deficiencies of in vitro experimental systems. The enzyme preparations from mammalian cells are added to in vitro systems for simulating in vivo xenobiotic bioactivation [8]. Subcellular preparations are obtained by the centrifugation of tissue homogenates. Any tissue could be applied [18]. However, liver is the most widely used because the enzymes from the cytochrome P450 system are located mainly in the endoplasmic reticulum of hepatic cells. There are two types of subcellular preparations. On the one hand, S9 fraction is obtained with one centrifugation step at 9000 *g*; it contains the microsomal and the cytosolic phases. The microsomal fraction requires an ultracentrifugation at 100,000 *g* to separate the cytosolic soluble phase from the microsomes [18]. The most widely used S9 fraction is obtained from rodent livers treated with inducing agents [8]. Liver homogenates were recommended as the most convenient tissue in the Ames test [19]. When validating the SOS assays as screening tests, liver S9 was also the most widely used [15,20,21,22,23,24,25]. However, some procarcinogens could need another type of metabolic preparation to show their biological effects [4,5]. For example, an Ames protocol modification was the inclusion of a hamster S9 fraction for the assessment of azo compounds [26,27,28]. In addition, the use of a different organ other than liver for preparing the S9 fraction may be recommended in some circumstances. For instance, OTA was assessed with a mice kidney microsomal fraction because the kidney is the target organ of OTA genotoxicity in rodents [14]. Moreover, the kidney is one of the tissues responsible of the metabolic conversion of fusarenon-X into nivalenol [29] and T-2 toxin transformation into HT-2 [30,31]. The present work analyzes the mutagenic potential of 12 mycotoxins with a standard liver S9 fraction and a kidney S9 fraction with the SOS/umu test. Most of the mycotoxins are assessed with kidney S9 and the SOS/umu test for the first time here. This assay is usually performed once in the context of drug screening, but in the present study, 2–4 independent experiments have been carried out for confirming the results and analyzing the variability of the test.

4NQO is standardly used as positive control without metabolic activation [32]. According to our results, it had a weak genotoxic response in this condition. However, it was genotoxic with both metabolic fractions, which was not expected. Moreover, the genotoxicity with kidney S9 was greater than without metabolic activation. 4NQO is quickly reduced into 4-hydroxyaminoquinolone 1-oxide (4HAQO), a carcinogenic metabolite [33]. The suspected enzyme responsible of the reduction was located in the supernatant fraction of rat liver and kidney, but with a higher specific activity in liver [34]. However, we registered greater genotoxicity with kidney S9. The carcinogenic activity of 4HAQO was higher than 4NQO in mice. Consequently, it was suggested that 4NQO acts through its reduced form, 4HAQO [34]. Therefore, in our case, 4NQO’s metabolic conversion with both S9 fractions and the possible presence of 4HAQO could explain the genotoxic response obtained with metabolic activation.

2AA is probably the most widely used mutagen [35] and seems to be activated by hepatic preparations from most animal species (rat, mice, hamster, pig and human) [36], which agrees with the results obtained with liver S9. However, it was negative with kidney S9. Just one author assessed the mutagenicity of 2AA with the Ames test and kidney S9 from oyster toadfish. The addition of the kidney S9 increased the mutagenicity of 2AA [37]. The different species and S9 concentration in the metabolic activation mixture could be the cause of the discrepancy.

AFB1 is a genotoxic and carcinogenic compound. It forms DNA adducts and induces gene mutations and chromosomal aberrations. The mutagenicity in *Salmonella* tester strains TA 98 and TA 100 was 1000 times higher with metabolic activation than without bioactivation [38]. AFB1 has been used as positive control for liver S9 in SOS test [32,39,40]. The results with metabolic activation in our study are also greater than without, with a more pronounced response with liver S9 than with kidney S9. Just one author explored the mutagenicity of AFB1 with the Ames test and kidney S9 from oyster toadfish. The addition of the kidney S9 increased the mutagenicity of AFB1 [37].

STER is an intermediate of the aflatoxins biosynthesis pathway with a comparable genotoxic potential than AFB1 [41]. Moreover, STER produced a greatest response than AFB1 in SOS/umu test with liver microsomes from human and rat [42]. In most of the SOS tests it is also genotoxic [40,43]. In our study STER was positive with liver S9 but equivocal with kidney S9. In all cases, it was less genotoxic than AFB1. Nonetheless, the assessment with kidney S9 has been conducted for the first time.

Regarding trichothecenes, NIV, F-X, DON, 3ADON and T-2 were assessed with the Ames test without and with liver S9. In all cases, mutagenic activity was not found [44,45,46,47,48,49]. DON was also negative with SOS chromotest [45]. In the present study, all were negative without and with liver S9. In some cases, the genotoxicity assessment with SOS/umu test was carried out for the first time (15ADON and HT-2) [50,51]. Moreover, the genotoxicity assessment with S9 fraction of kidney origin was carried out for the first time for all trichothecenes. DON and F-X were also negative with kidney S9; however, NIV, 3ADON, 15ADON, T-2 and HT-2 produced equivocal results.

Ochratoxin A is suspected of being one of the etiological agents of Balkan Endemic Nephropathy (BEN) and was associated with urinary track tumors in humans [52]. Since its last revision in 2020, the uncertainty of the mode of action for kidney carcinogenicity has increased [13]. However, it is known that oxidative stress is involved [53]. OTA mutagenicity assessed with *Salmonella* tester strains was consistently negative [13], which agrees with our results obtained in all conditions. OTA induced a weak response in SOS chromotest, but without a concentration-response trend, which was considered a negative outcome [40]. OTA induced SOS repair activity with SOS chromotest without metabolic activation. However, the concentration tested was cytotoxic [54]. A mixture of OTA and OTB (85% and 15%) induced a weak mutagenic response with a liver S9 fraction in the Ames test. However, in the same experiment, OTA alone was negative [48]. Therefore, in most cases, OTA was found as negative with and without metabolic activation in the SOS test [43,55], which agrees with our results.

However, OTA was mutagenic when using a mice renal microsomal fraction in 1535, 1538 and 98 strains with the Ames test. NADP and arachidonic acid were used as cofactors, and in both cases, OTA was positive in the three strains [14]. The type of preparation (microsomal fraction) and the species (mice) are different from our study, as is the use of arachidonic acid, which could explain the different results obtained with the kidney fraction in the present work. The mutagenicity of 1535 was higher with this cofactor, which would support the hypothesis according to which prostaglandin synthase would be the key element for oxidative metabolism of genotoxic compounds in mouse kidney [14].

Regarding zearalenone, negative results were observed in the Ames test without and with (liver S9) metabolic activation [44,48,56]. In addition, negative outcomes were obtained with SOS tests [39,40,43,55,57], which agrees with our results. In just one case, ZEA was able to induce SOS repair in *Escherichia coli*, but the genotoxic concentration (1.5 mM) was also toxic (IC50 1.45 mM) [58]. The assessment with kidney S9 has been performed for the first time.

FB1 was related with renal carcinogenicity in rats, probably not mediated by a genotoxic mode of action [59]. The mutagenic potential studied with the Ames test was negative without and with liver S9 [45,60,61]. Knasmüller et al. 1997 [45] assessed FB1 genotoxicity with *Escherichia coli PQ37* in the SOS chromotest and was found to be negative, which is in agreement with our results [45]. In the present study, FB1 reached IF values of 1.99 with kidney S9 (31 and 63 µg/mL in day 1) but without a dose–response tendency (see Figure S6). The genotoxicity assessment with kidney S9 has been performed for the first time.

In all compounds tested, the IF variability was high (CV > 30%). In addition, it was higher with kidney S9 than with liver S9, except with NIV and AFB1, which showed higher variability with liver S9 than with kidney S9. In spite of this, the SOS/umu test can be considered a reproducible assay. This is very evident with the positive controls results. In seven out of eight experiments, it could be concluded that 4NQO was genotoxic with kidney S9, and in eight out of eight experiments, it was genotoxic with PBS. For 2AA, in eight out of eight experiments, it was genotoxic with liver S9. AFB1, was also genotoxic in all experiments with liver S9 and kidney S9. The common aspect observed in all of them was the concentration–response tendency. For NIV, 3ADON and T-2, IF values above two were registered in three out of four experiments with kidney S9; 15ADON and HT-2 in two out of four experiments; and STER in one out of four experiments. However, a concentration–response tendency was not observed in any of those cases. It seems that the high variability with kidney S9 would affect the reproducibility of these compounds under this condition. Therefore, these mycotoxins were classified as equivocal. In summary, the recommendation could be, depending on the compound, that the assay could be performed once, e.g., if there is a clear positive (with a concentration-response tendency) or negative response (IF values < 1.5). However, when analyzing equivocal compounds (IF values around 2), it could be advisable to perform at least two independent experiments. In addition, the Ames test is the gold standard assay for mutagenicity screening [62]. The SOS/umu test has a high concordance with this assay [15,16]. Therefore, it would be advisable to obtain confirmatory results with the miniatured version of the Ames test using a kidney S9 of the mycotoxins with equivocal results obtained in this work with kidney S9 (sterigmatocystin and trichothecenes type A and B). The miniaturized version of the Ames test has a high concordance with the standard version [63]. Only in the case of still obtaining equivocal results could the standard version be performed.

## 4. Conclusions

The SOS/umu test can be considered a reproducible test. Consistent results have been obtained with negative (PBS) and positive (4NQO and 2AA) controls. Thus, this assay could be used not only for screening but also for genotoxic characterization as a first step. The organ used for the S9 fraction preparation has an influence on the genotoxic activity of some compounds, including 4NQO and 2AA and some mycotoxins. Thus, the use of S9 fraction from kidney tissue may be advisable in some cases.

## 5. Materials and Methods

### 5.1. Chemicals and Reagents

DMSO and Na₂CO₃ were purchased from PanReac AppliChem (Barcelona, Spain). Bactotryptone for TGA medium was obtained from Bectone Dickinson (Madrid, Spain) and dextrose and NaCl were from PanReac AppliChem (Barcelona, Spain). Ampicillin, ONPG (2-nitrophenyl- β-D-galactopyranoside), the B-buffer ingredients (Na₂HPO₄ 2H₂O, NaH₂PO₄ H₂O, MgSO₄ 7H₂O, sodium dodecyl sulfate, β-mercaptoethanol) in which the ONPG was dissolved, PBS ingredients (Na₂HPO₄ 2H₂O, NaH₂PO₄ H₂O) and the positive controls 2-aminoanthracene (2AA) and 4-nitroquinoline-N-oxide (4NQO) were purchased from Sigma-Aldrich (Darmstadt, Germany). The KCl for B-buffer was from PanReac AppliChem (Barcelona, Spain). Ingredients for the S9 mix preparation were obtained from Sigma-Aldrich (Darmstadt, Germany)—phosphate buffer (NaH₂PO₄ H₂O, Na₂HPO₄ 2H₂O), glucose-6-phosphate and NADP solutions—or from PanReac AppliChem (Barcelona, Spain)—saline solution (MgCl₂ 6H₂O, KCl).

### 5.2. Experimental System

The genetically modified *Salmonella typhimurium* 1535/pSK1002 used in the SOS/umu test was purchased from the German Collection for microorganisms and Cell cultures (DSMZ 9274) (Berlin, Germany). The bacterium contains a plasmid in which two genes are fused, one involved in DNA repair and the other accountable for β-galactosidase activity. Therefore, SOS repair activity was monitored through β-galactosidase induction activity determined with spectrophotometric measures.

### 5.3. Mycotoxins

All mycotoxins were purchased in powder form from Sigma Aldrich (Darmstadt, Germany), dissolved in DMSO or water and maintained at −20 °C until use. The reference commercial number and CAS number is indicated in each case: AFB1 (A6636; CAS: 1162-65-8), STER (S3255; CAS:10048-13-2), DON (D0156; CAS: 51481-10-8), F-X (33438; CAS:23255-69-8), NIV (32929; CAS: 23282-20-4), 3ADON (A6166; CAS:50722-38-8), 15ADON (32928; CAS: 88337-96-6), T-2 toxin (33947; CAS: 21259-20-1), HT-2 toxin (T4138; CAS: 26934-87-2), OTA (O1877; CAS: 303-47-9), ZEA (Z2125; CAS: 17924-92-4) and FB1 (F1147; CAS: 116355-83-0).

### 5.4. Rat Tissue Fractions and S9 Mix Preparation

Two rat S9 fractions were used, one obtained from liver and the other one from kidney. The kidney S9 was purchased from Tebu-bio (Barcelona, Spain), and the liver S9 was obtained from Trinova Biochem (Giessen, Germany). Both were extracted from Sprague Dawley male rats of 5 to 8 weeks of age. Liver S9 was obtained from rats treated with Aroclor 1254 as enzyme inductor. The kidney S9 was extracted from rats not treated with any inducing agent. The sterility of kidney S9 fraction was verified in our laboratory.

The S9 mixtures at 8% with each one of the fractions were prepared just before the assay by adding the S9 fraction to the following solution: phosphate buffer (0.2 M pH 7,4 NaH₂PO₄ H₂O, Na₂HPO₄ H₂O), glucose-6-phosphate (1 M), NAPD (0.1 M) and saline solution (1.6 M MgCl₂ 6H₂O, 0.4 M KCl). The S9 mixture was filtered through a 0.45 µ filter.

### 5.5. Replicates and Number of Independent Experiments

The number of independent experiments for the positive and negative controls was 8 in all conditions. The number of replicates for the negative control in each experiment was 7. In the case of positive controls and mycotoxins, technical replicates were not included in each experiment. The number of independent experiments for the mycotoxins are between 2 and 4. If the conclusion among the experiments was the same (no genotoxic/genotoxic), there were two independent experiments. If there was no concordance in the conclusion of the experiment, it was repeated up to 4 times. Some experiments of the PBS and liver S9 fraction conditions were already published [3] (day 1 of FB1; day 3 of AFB1, OTA, ZEA, DON and F-X; day 4 of STER; days 2 and 3 of NIV, 3ADON, 15ADON, T-2 and HT-2).

### 5.6. SOS/umu Assay

The SOS/umu test was performed according to the method proposed by [20,24], with some modifications. The procedure of the assay and the stock solution preparation of the mycotoxins and positive controls are described in [3].

The bacteria were incubated overnight at 37 °C in 100 mL TGA medium supplemented with ampicillin (50 µg/mL), with slight orbital shaking (155 rpm) from 15 to 17 h until an optimal orbital density (OD₆₀₀ from 0.5 to 1.5) was reached. Then, the overnight culture was diluted with fresh TGA medium (not supplemented with ampicillin) and incubated for 2 h at 37 °C with orbital shaking (155 rpm) in order to obtain a log-phase bacterial growth culture (OD₆₀₀ from 0.15 to 0.4).

The test was performed in the absence (PBS) and presence of an external metabolic activation system (8% of rat S9 mix from liver or kidney S9 fraction). In each test, negative and positive controls were included.

The test procedure was as follows: first, mycotoxins were dissolved at their respective maximum concentrations. Then, 11 serial half dilutions in DMSO (or water for FB1) of mycotoxins and positive controls were prepared in a 96-well plate (plate A). The final volume in each well was 10 µL. The wells on the last row of the plate contained the negative control (DMSO or water). Afterwards, 70 µL water was added to each well. At this point, each well was checked in order to detect any precipitation of the mycotoxins. Then, the S9 mixes were prepared.

Thereafter, in another 96-well plate (plate B), 10 µL S9 mix or 10 µL PBS, were added, followed by 25 µL of the concentrations of the different mycotoxins previously prepared (plate A). Finally, 90 µL/well of exponentially growing bacteria suspension was added to each well. Then, the plates were incubated for 4 h at 37 °C with orbital shaking (500 rpm). After the incubation period, A₆₀₀ was measured to evaluate toxicity as follows:

(1) $$\%\;\mathrm{Survival}=\frac{A_{600}\;\mathrm{for}\;\mathrm{each}\;\mathrm{concentration}\;\mathrm{tested}\;}{\mathrm{Average}\;A_{600}\;\mathrm{for}\;\mathrm{negative}\;\mathrm{control}\;}\;\times \;100$$

Afterwards, for the determination of β-galactosidase activity, 30 µL/well of treatment plates (plates B) were transferred to new wells (plates C) containing 150 µL/well of ONPG solution for the enzymatic reaction. A total of 0.9 mg/mL in B-buffer was prepared according to [24] for an enzymatic reaction. Plates C were incubated for 30 min at 28 °C with orbital shaking (500 rpm) in the dark. After the incubation period, the reaction was stopped by adding 120 µL/well of Na₂CO₃ (1M). Finally, A₄₂₀ was measured and β-galactosidase activity was determined as follows:

β-galactosidase activity relative units (RU):

(2) $$\mathrm{RU}=\frac{A_{420}\;\mathrm{for}\;\mathrm{each}\;\mathrm{concentration}\;\mathrm{tested}\;}{A_{600}\;\mathrm{for}\;\mathrm{each}\;\mathrm{concentration}\;\mathrm{tested}\;}$$

Additionally, induction factor (IF)

(3) $$\mathrm{IF}=\frac{\mathrm{RU}\;\mathrm{for}\;\mathrm{each}\;\mathrm{concentration}\;\mathrm{tested}\;}{\mathrm{Average}\;\mathrm{RU}\;\mathrm{for}\;\mathrm{negative}\;\mathrm{control}\;}$$

where average β-galactosidase RU for negative control:

(4) $$\mathrm{RU}=\frac{\mathrm{Average}A_{420}\;\mathrm{for}\;\mathrm{negative}\;\mathrm{control}\;}{\mathrm{Average}\;A_{600}\;\mathrm{for}\;\mathrm{negative}\;\mathrm{control}\;}$$

IF values above or equal to two were considered as positive when the bacterial survival is above 80%, following the criteria of [20]. IF values below 1.5 were considered negative. Finally, IF among 2 and 1.5 were considered equivocal.

## Data Availability

Data are contained within the article.

## Supplementary Materials

The following are available online at https://www.mdpi.com/article/10.3390/toxins14060400/s1, Figure S1. AFB1 and STER results in the SOS/umu test without metabolic activation (PBS). Figure S2. DON and F-X results in the SOS/umu test without metabolic activation (PBS) (*n* = 3 experiments). Figure S3. DON and F-X results in the SOS/umu test with metabolic activation from liver S9 or kidney S9 (*n* = 2 experiments). Figure S4. NIV, 3ADON, 15ADON, T-2 and HT-2 results in the SOS/umu test without metabolic activation (PBS) (*n* = 4 experiments). Figure S5. OTA, ZEA and FB1 results in the SOS/umu test without metabolic activation (PBS). Figure S6. OTA, ZEA and FB1 results in the SOS/umu test with metabolic activation from liver S9 and kidney S9 (*n* = 2 experiments).
