# Supplementary material for: Genotoxicity of 12 Mycotoxins by the SOS/umu Test: Comparison of Liver and Kidney S9 Fraction
Source: Toxins (Basel). 2022 Jun 10;14(6):400. doi: 10.3390/toxins14060400 (PMC9228656; doi:10.3390/toxins14060400)
Supplement: Supplementary file 1 [file toxins-14-00400-s001.zip › toxins-1754449-supplementary.pdf]

toxins

MDPI

Article

Genotoxicity of 12 Mycotoxins by the SOS/umu Test: Comparison of Liver and Kidney S9 Fraction

María Alonso-Jauregui [1], Elena González-Peñas [2], Adela López de Cerain [1] and Ariane Vettorazzi [1,*]

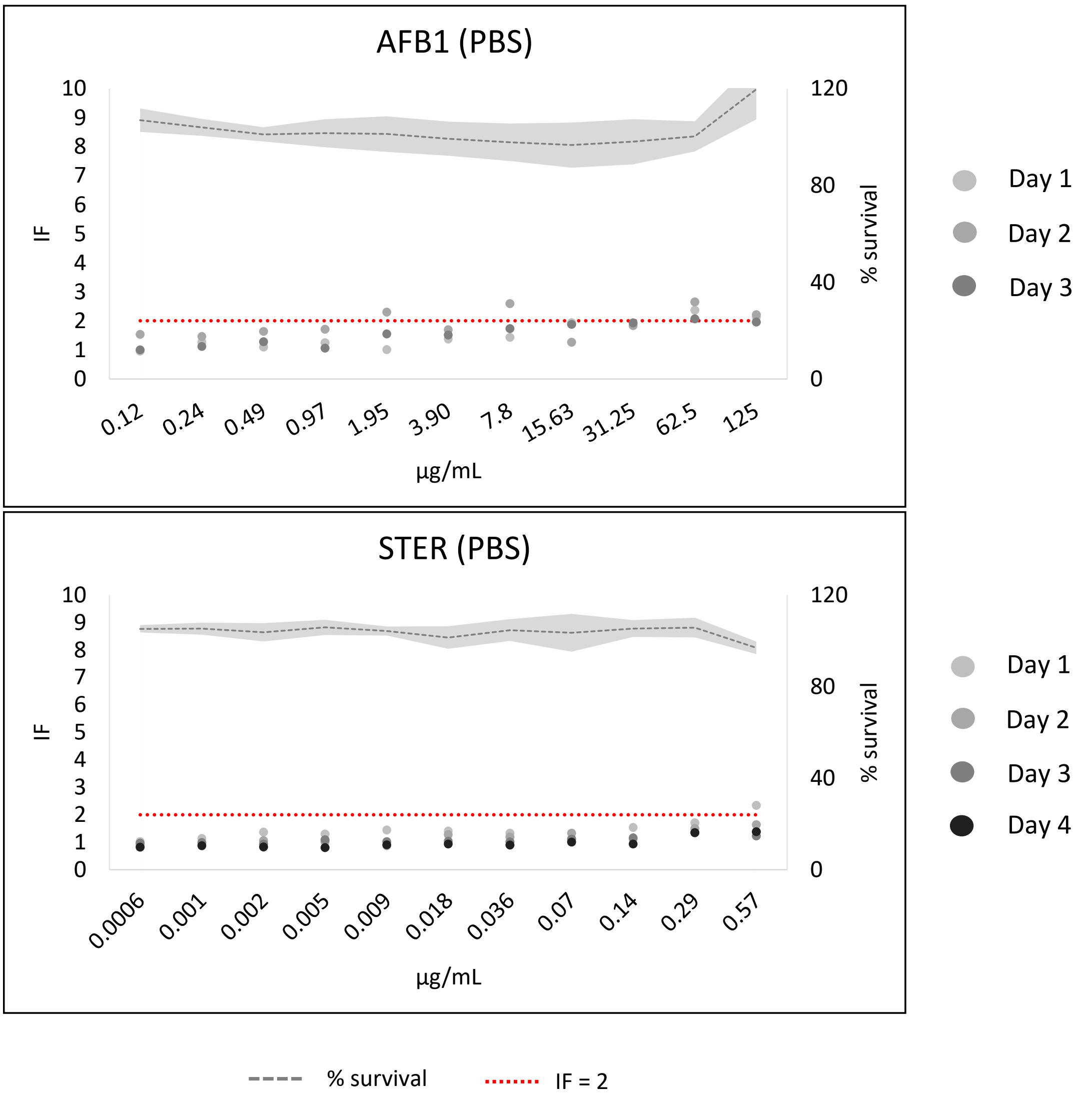

Figure S1. AFB1 and STER results in the SOS/umu test without metabolic activation (PBS). The number of experiments of AFB1 is n=3. The number of experiments of STER is 4. The dots represent the inductor factor (IF) of each individual experiment and the grey line the mean bacterial survival as percentage. The standard deviation of the survival is presented with the soft grey area. Concentrations were considered non-toxic if survival was > 80%. A compound was considered genotoxic if inductor factor (IF) value was ≥ 2 at non-toxic concentrations. The red line has been depicted to indicate IF=2. Data from two assays (days 3 (AFB1) and 4 (STER)) were published in [3].

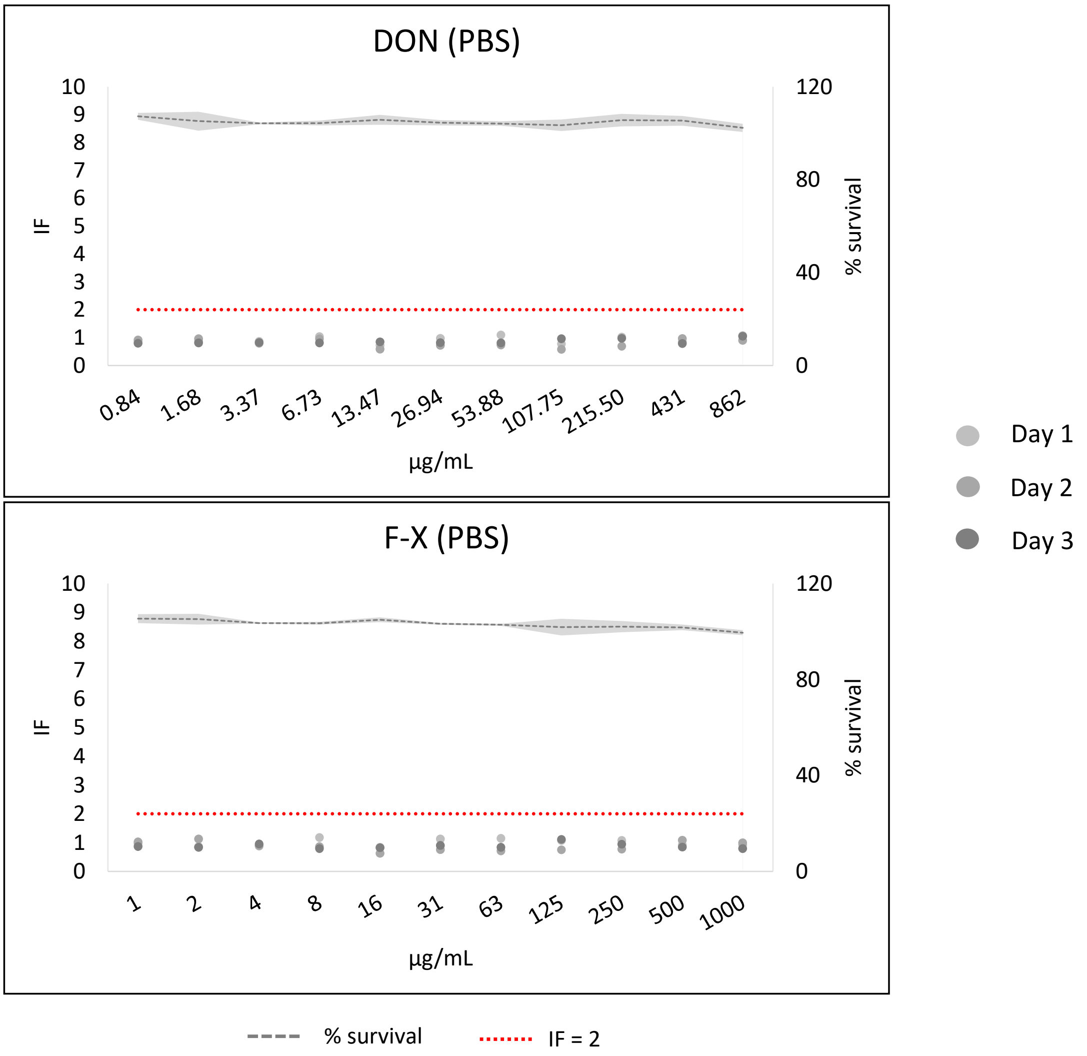

Figure S2. DON and F-X results in the SOS/umu test without metabolic activation (PBS) (n=3 experiments). The spots represent the inductor factor (IF) of each individual experiment and the grey line the mean bacterial survival as percentage. The standard deviation of the survival is presented with the soft grey area. Concentrations were considered non-toxic if survival was > 80%. A compound was considered genotoxic if inductor factor (IF) value was ≥ 2 at non-toxic concentrations. The red line has been depicted to indicate IF=2. Data from day 3 was published in [3].

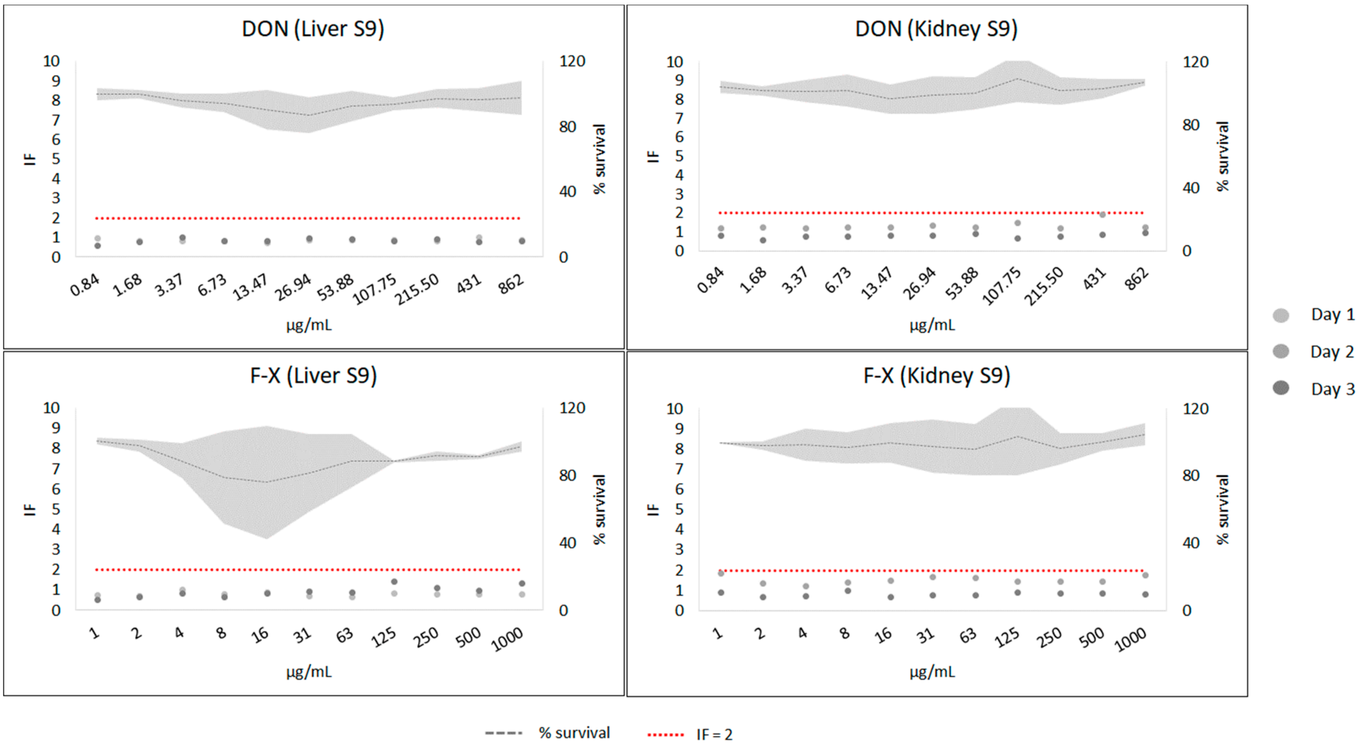

Figure S3. DON and F-X results in the SOS/umu test with metabolic activation from liver S9 or kidney S9 (n=2 experiments). The dots represent the inductor factor (IF) of each individual experiment and the grey line the mean bacterial survival as percentage. The standard deviation of the survival is presented with the soft grey area. Concentrations were considered non-toxic if survival was > 80%. A compound was considered genotoxic if inductor factor (IF) value was ≥ 2 at non-toxic concentrations. The red line has been depicted to indicate IF=2. Data from day 3 was published in [3]. % survival values above 120% were corrected to 120%.

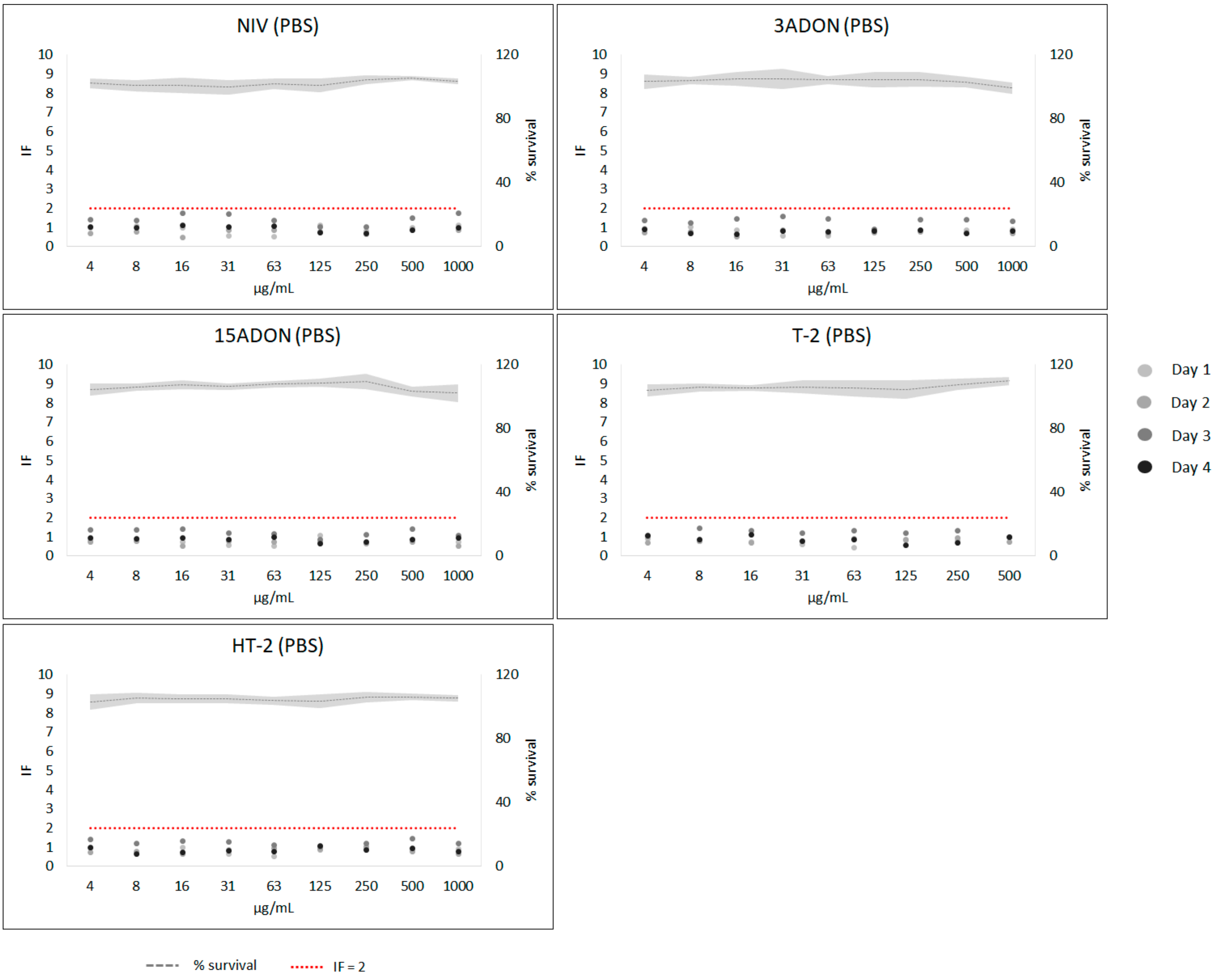

Figure S4. NIV, 3ADON, 15ADON, T-2 and HT-2 results in the SOS/umu test without metabolic activation (PBS) (n=4 experiments). The dots represent the inductor factor (IF) of each individual experiment and the grey line the mean bacterial survival as percentage. The standard deviation of the survival is presented with the soft grey area. Concentrations were considered non-toxic if survival was > 80%. A compound was considered genotoxic if inductor factor (IF) value was ≥ 2 at non-toxic concentrations. The red line has been depicted to indicate IF=2. Data from two assays (days 2 and 3) were published in [3].

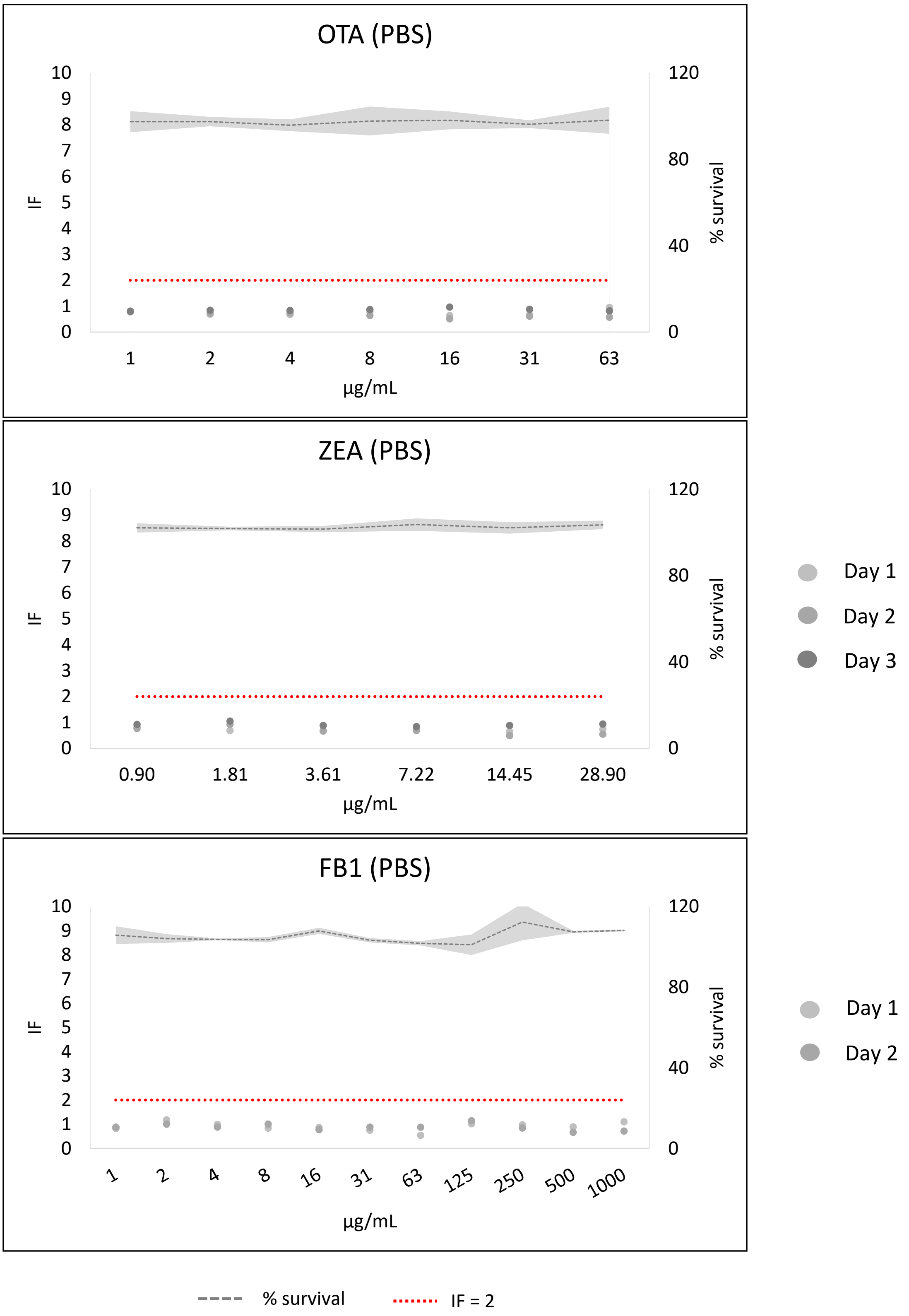

Figure S5. OTA, ZEA and FB1 results in the SOS/umu test without metabolic activation (PBS). The number of experiments for OTA and ZEA are n=3 and for FB1 are n=2. The dots represent the inductor factor (IF) of each individual experiment and the grey line the mean bacterial survival as percentage. The standard deviation of the survival is presented with the soft grey area. Concentrations were considered non-toxic if survival was > 80%. A compound was considered genotoxic if inductor factor (IF) value was ≥ 2 at non-toxic concentrations. The red line has been depicted to indicate IF=2. Data from two assays (days 1 (FB1) and 3 (OTA, ZEA)) were published in [3].

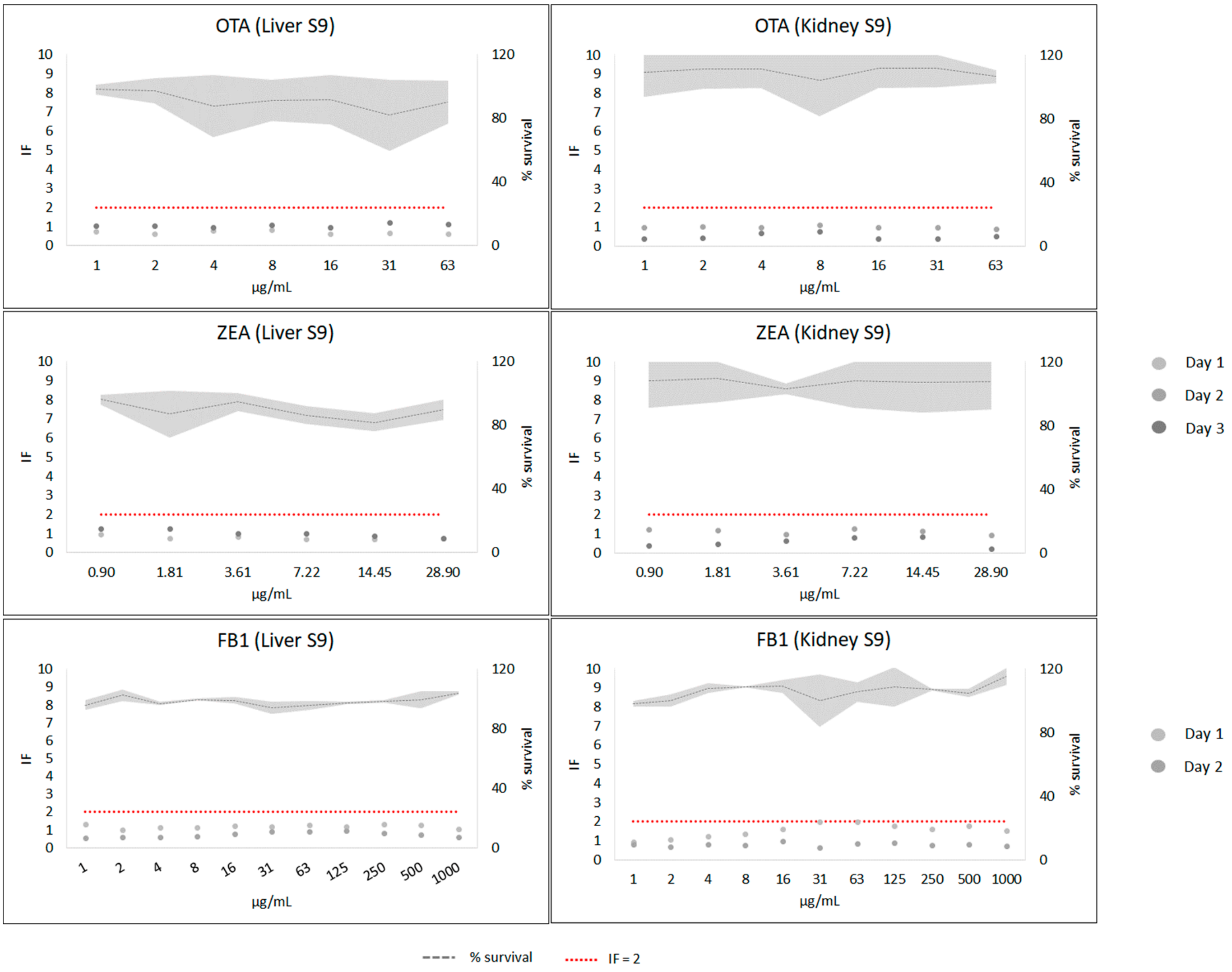

Figure S6. OTA, ZEA and FB1 results in the SOS/umu test with metabolic activation from liver S9 and kidney S9 (n=2 experiments). The dots represent the inductor factor (IF) of each individual experiment and and the grey line the mean bacterial survival as percentage. The standard deviation of the survival is presented with the soft grey area. Concentrations were considered non-toxic if survival was > 80%. A compound was considered genotoxic if inductor factor (IF) value was ≥ 2 at non-toxic concentrations. The red line has been depicted to indicate IF=2. Data from two assays (days 1 (FB1) and 3 (OTA, ZEA)) were published in [3].
